# The Critical Role of Enhanced OXPHOS and Mitochondrial Hyperpolarization in Simulated Microgravity‐Induced Oocyte Maturation Arrest

**DOI:** 10.1002/advs.202505570

**Published:** 2025-07-18

**Authors:** Lei Ge, Yuqing Gao, Feifei Du, Chiyuan Ma, Tianxia Xiao, Yali Yang, Xiaohua Lei, Jian V. Zhang

**Affiliations:** ^1^ Center for Energy Metabolism and Reproduction Shenzhen Institutes of Advanced Technology Chinese Academy of Sciences Shenzhen Guangdong 518055 China; ^2^ University of Chinese Academy of Sciences Beijing 100049 China; ^3^ Shenzhen Key Laboratory of Metabolic HealthShenzhen Metabolism and Reproductive Targeted Delivery Proof‐of‐Concept Center Shenzhen Institutes of Advanced Technology Shenzhen Guangdong 518055 China; ^4^ Department of Biomedical Sciences Faculty of Health Sciences University of Macau Macau 999078 China; ^5^ Faculty of Pharmaceutical Sciences Shenzhen University of Advanced Technology Shenzhen Guangdong 518028 China; ^6^ Sino‐European Center of Biomedicine and Health Shenzhen Guangdong 518000 China

**Keywords:** meiotic progression, microtubule‐organizing center, M‐phase extension, oxidative phosphorylation, simulated microgravity

## Abstract

Meiosis is essential for sexual reproduction, yet the impact of microgravity on oocyte maturation remains unclear, raising concerns for reproductive success in space environments. Here, it is examined the effects of simulated microgravity (SMG) on mouse oocytes and found that SMG impaired mitochondrial function, evidenced by elevated oxidative phosphorylation and mitochondrial membrane hyperpolarization, resulting in meiotic arrest. This response is distinct from that induced by other stressors or seen in somatic cells under microgravity, highlighting the unique sensitivity of oocytes. SMG also caused mitochondrial mislocalization, which activated the unfolded protein response and suppressed mitochondrial gene expression. Despite accelerating meiotic progression, SMG delayed microtubule‐organizing center (MTOC) coalescence. This misalignment led to spindle defects, reduced polar body extrusion, and increased aneuploidy, compromising oocyte quality. The spindle assembly checkpoint (SAC) remained functional, suggesting mitochondrial dysregulation‐not SAC failure‐drives meiotic acceleration. Notably, even oocytes that reached maturation under SMG exhibited polarity loss and reduced developmental potential. Extending metaphase I by inhibiting the anaphase‐promoting complex rescued MTOC assembly and spindle formation, significantly improving maturation rates. These findings identify mitochondrial dysfunction as a key mediator of SMG‐induced meiotic failure and propose M‐phase regulation as a strategy to safeguard female fertility in space environments.

## Graphical abstract

Simulated microgravity induces distinct mitochondrial hyperactivation patterns‐elevated OXPHOS and MMP‐which accelerate meiosis without SAC failure but delays MTOC coalescence, leading to spindle defects and meiotic arrest. Prolonging M‐phase restores MTOC assembly and oocyte maturation.

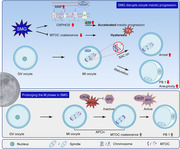

## 1 Introduction

Germ cells are continuously exposed to mechanical stimuli from gravitational forces, cell‐extracellular matrix interactions, and cell‐cell communication.^[^ ^1^, ^2^ ^]^ These forces are essential for morphogenesis, shaping biological structures and regulating cellular behaviors. However, most studies on these processes are conducted under Earth's constant gravitational pull, leaving a gap in our understanding of how altered gravity affects cellular physiology. As space exploration advances, understanding the biological effects of microgravity has become increasingly important for human health.

Over six decades of manned space missions have revealed significant physiological changes induced by microgravity, such as bone loss, muscle atrophy, and cardiovascular remodeling. Despite these insights, the effects of microgravity on mammalian reproduction‐particularly oocyte maturation and meiosis‐remain poorly understood. While non‐mammalian species can reproduce in space,^[^ ^3^, ^4^ ^]^ mammalian reproduction is far more complex, involving tightly regulated processes sensitive to environmental changes. Despite numerous experiments, successful mammalian reproduction in space has not been achieved. Previous studies have reported that female mice mated in orbit showed early signs of pregnancy post‐flight, but embryonic development failed to progress,^[^ ^5^ ^]^ suggesting that key stages of mammalian reproduction, especially oocyte maturation, may be disrupted in microgravity. Our earlier work demonstrated that preimplantation mouse embryos can develop in space,^[^ ^6^ ^]^ further highlighting the sensitivity of reproductive processes to gravitational changes. However, the mechanisms through which microgravity affects mammalian oocyte meiosis remain unclear.

Meiosis is a tightly regulated process that ensures chromosome segregation, and errors can lead to aneuploidy, infertility, and developmental disorders,^[^ ^7^, ^8^ ^]^ The accurate progression of meiosis relies on the assembly of a microtubule‐based bipolar spindle, which ensures proper chromosome separation.^[^ ^9^ ^]^ The spindle assembly checkpoint (SAC) monitors chromosome attachment to the spindle, preventing anaphase onset until all chromosomes are correctly aligned.^[^ ^10^, ^11^ ^]^ Disruption of SAC function triggers the anaphase‐promoting complex (APC), leading to the degradation of securin and cyclin B, thereby promoting meiotic progression. Given that mammalian oocytes are particularly prone to chromosomal abnormalities,^[^ ^9^ ^]^ microgravity might significantly affect spindle assembly, chromosome alignment, and mitochondrial function during meiosis.

Understanding how microgravity affects oocyte maturation is essential for safeguarding female reproductive health during long‐term space missions. Oocyte development is regulated by integrated biochemical and mechanical signals that control cell behavior and differentiation. However, the signaling pathways and cellular responses involved in space microgravity remain largely unknown. In this study, we aimed to investigate how simulated microgravity (SMG) affects oocyte meiotic progression and whether prolonging the M phase via APC inhibition can mitigate these effects. Previous studies have shown that SMG disrupts intercellular communication in follicles,^[^ ^12^ ^]^ and impairs the early development of preantral follicles.^[^ ^13^ ^]^ Here, we optimized an oocyte culture platform under SMG conditions using the random positioning machine (RCCS) and employed single‐cell transcriptome analysis (Smart‐seq2) to investigate molecular changes during meiotic progression.

Our results demonstrate that SMG accelerates meiotic progression while delaying microtubule‐organizing center (MTOC) aggregation, leading to spindle defects, chromosome misalignment, and increased aneuploidy. SMG also disrupted mitochondrial function by increasing oxidative phosphorylation (OXPHOS) and mitochondrial membrane potential (MMP). Importantly, prolonging the M phase via APCin inhibition rescued spindle defects and maturation arrest under SMG conditions. These findings provide novel insights into the molecular mechanisms underlying oocyte meiotic disruption in microgravity and propose a potential strategy to mitigate reproductive risks during space missions by targeting meiotic progression. Understanding these mechanisms is essential for ensuring reproductive success and safeguarding astronaut health during long‐term space exploration.

## 2 Results

### 2.1 Impact of SMG on Oocyte Maturation, Meiotic Progression, and Developmental Potential

**Figure 1:**
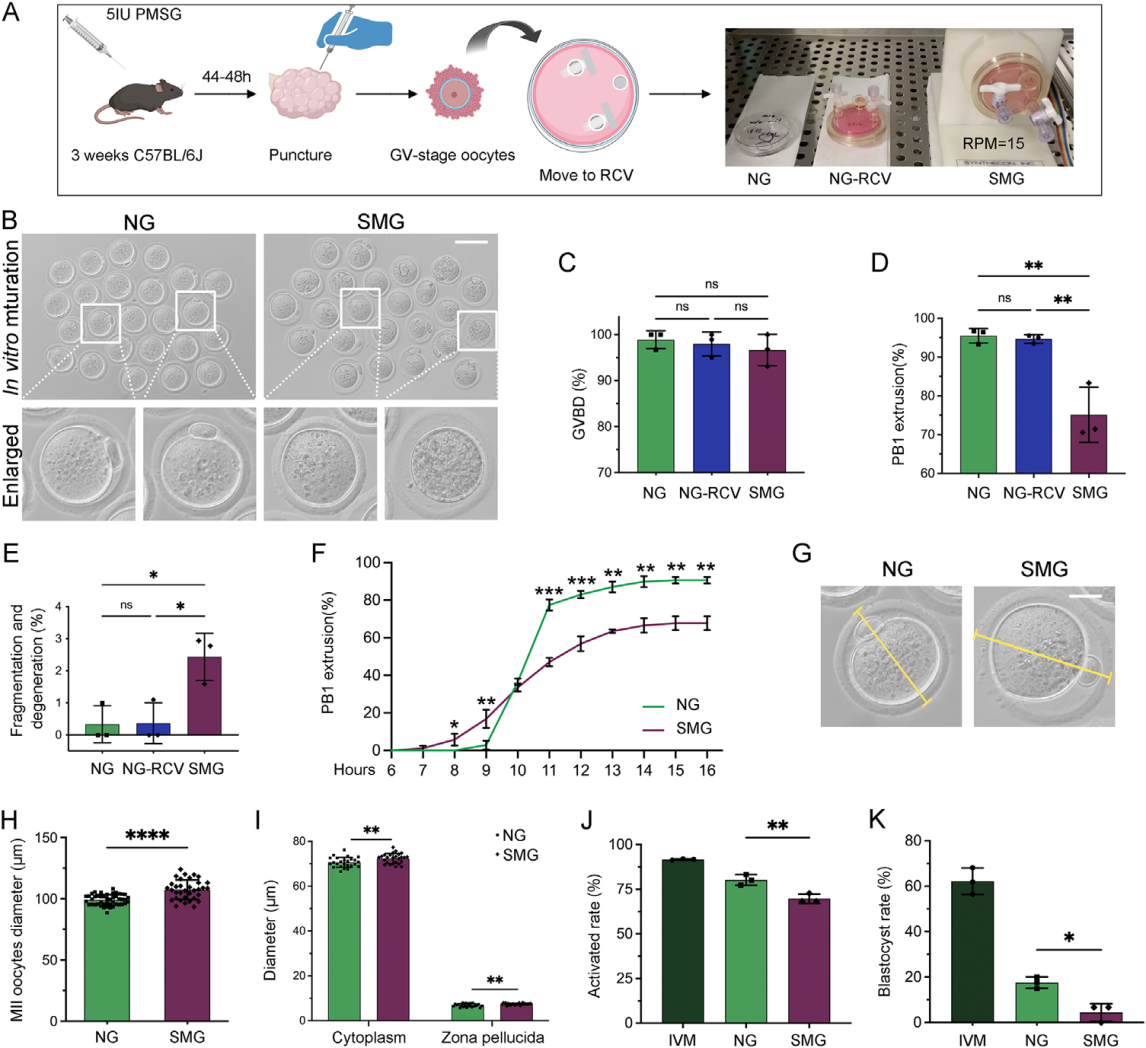
Simulated Microgravity (SMG) Impairs Oocyte Maturation and Developmental Potential. A) Schematic diagram of the experimental design. Three‐week‐old female mice underwent superovulation, and germinal vesicle (GV)‐stage oocytes were collected and cultured under three conditions: normal gravity (NG), NG with a rotary cell vessel (NG‐RCV), and SMG. B) Representative images of first polar body (PB1) extrusion in NG and SMG oocytes. Scale bar: 125 µm. C‐E) Quantitative analysis of GV breakdown (GVBD), PB1 extrusion, and oocyte fragmentation/degeneration rates across NG, NG‐RCV, and SMG groups. F) Time‐course analysis of PB1 extrusion rates at consecutive intervals to assess meiotic progression under different culture conditions. G, H) Representative images and measurements of metaphase II (MII) oocyte diameter (µm) in NG and SMG groups. Scale bar: 25 µm. I) Quantification of cytoplasm and zona pellucida thickness (µm) in NG and SMG oocytes. J, K) Parthenogenetic activation and subsequent embryonic development. The proportion of two‐cell embryos was calculated using the total number of MII oocytes as the denominator. The blastocyst rate was determined using the number of two‐cell embryos as the denominator.

To investigate the effect of simulated microgravity (SMG) on oocyte maturation, we exposed three‐week‐old female mice to superovulation and collected GV‐stage oocytes, which were then cultured under three conditions: normal gravity (NG), normal gravity with RCV dish (NG‐RCV), and SMG (15 rotations per min, **Figure** **1**A). The use of the RCV dish did not influence GVBD or PB1 emission, nor did it affect oocyte degeneration or fragmentation in the NG condition (Figure 1C–E, NG *v.s*. NG‐RCV). Under SMG conditions, GVBD remained unaffected (Figure 1B,C), but PB1 emission was significantly inhibited (Figure 1B,D), and oocyte degeneration and fragmentation rates were notably higher in the SMG group (Figure 1E). Therefore, the NG group was selected as the control for subsequent experiments. We analyzed meiotic progression by refining the time points for PB1 emission. After 8 h of meiosis, the NG group had not yet emitted PB1, whereas SMG exposure significantly accelerated PB1 emission. The maximum difference between groups occurred at the 9‐h mark, with the NG group catching up after 10 h (Figure 1F). And the NG group consistently exhibited a higher PB1 emission rate thereafter (Figure 1F).

Examination of MII‐stage oocytes revealed that SMG exposure led to a significant increase in oocyte diameter compared to controls (Figure 1G,H). Additionally, the cytoplasmic and zona pellucida thicknesses were markedly increased in the SMG group (Figure 1I). Parthenogenetic activation experiments showed that the activation and embryonic development rates of mature oocytes in the NG group were normal, confirming the stability of the parthenogenetic system. However, both activation and embryonic development rates were significantly lower in the SMG group (Figure 1J,K). In conclusion, SMG exposure accelerates meiotic progression, disrupts oocyte maturation, and diminishes developmental potential.

### 2.2 SMG Disrupts Spindle Assembly and Delays MTOC Aggregation in Oocytes

**Figure 2:**
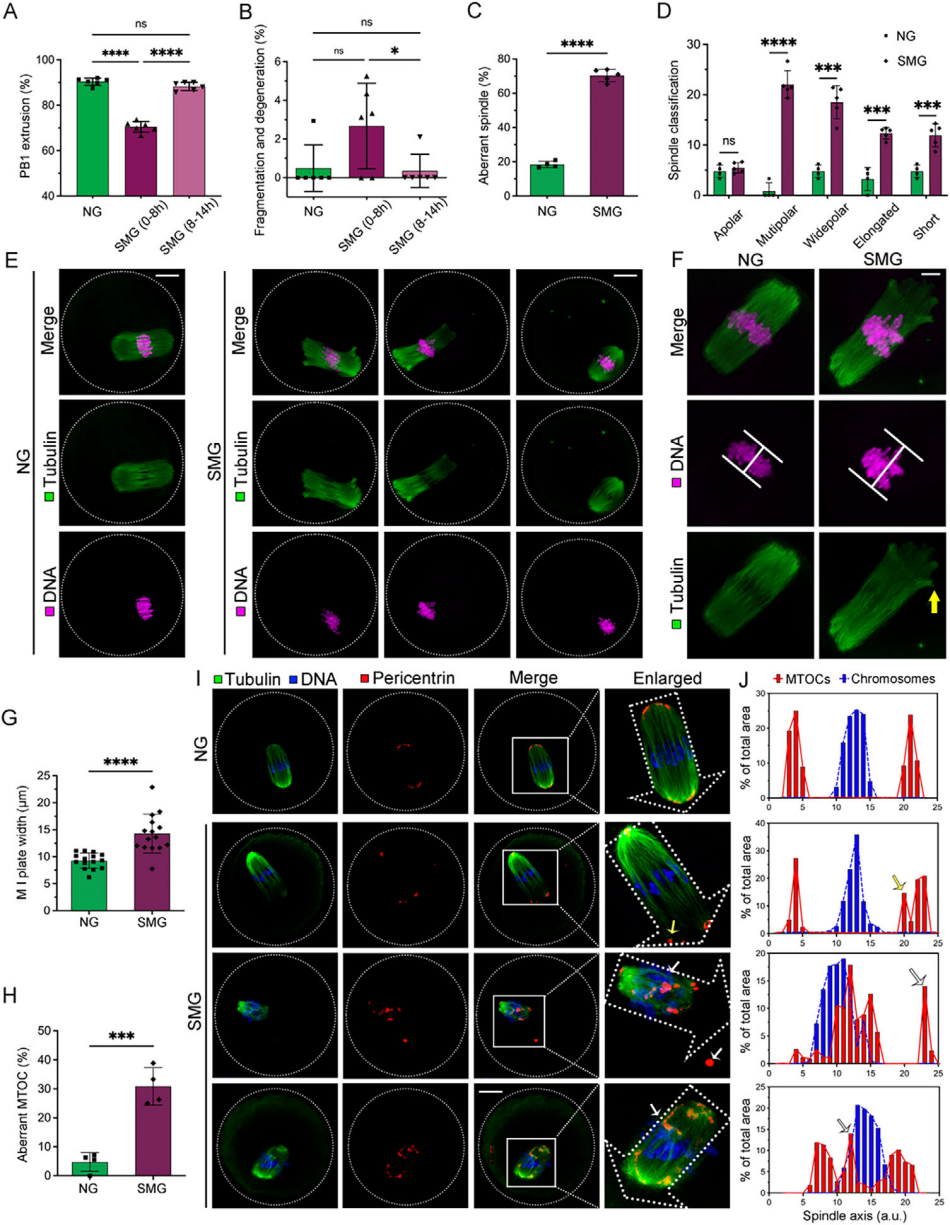
Effect of SMG on Spindle Assembly, Chromosome Alignment, and MTOC Organization in Mouse Oocytes. A, B) Quantitative analysis of PB1 extrusion and oocyte fragmentation/degeneration rates in NG, SMG (0–8 h), and SMG (8‐14 h) groups. C) Proportion of abnormal spindles in metaphase I (MI) oocytes cultured under NG and SMG conditions. D) Classification of spindle defects, including apolar, multipolar, wide polar, elongated, and short spindles, recorded in NG (n = 88) and SMG (n = 130) oocytes. E) Representative images of spindle morphology and chromosome alignment in MI oocytes. Oocytes were immunostained with anti‐α‐tubulin‐FITC to visualize spindles and counterstained with Hoechst to detect chromosomes. Scale bar: 25 µm. F, G) Representative images and quantification of chromosome plate width at the MI stage, recorded in NG (n = 15) and SMG (n = 15) oocytes. Scale bar: 5 µm. (H) Frequency of aberrant MTOC distribution in MI oocytes. I) Immunofluorescence images of pericentrin‐labeled MTOCs in MI oocytes from NG and SMG groups, recorded in NG (n = 58) and SMG (n = 74) oocytes. Scale bar: 25 µm. J) Quantitative analysis of MTOC distribution along the spindle axis in MI oocytes. Pericentrin signals were binarized to measure individual MTOC areas (arbitrary units), and their coordinates were plotted along the spindle axis (0‐25). MTOCs are shown in red, chromosomes in blue.

We cultured oocytes at different stages of meiosis under SMG conditions to explore the specific effects of SMG on spindle assembly and MTOC aggregation. Oocytes exposed to SMG prior to metaphase I (MI) (0–8 h) exhibited significantly reduced PB1 emission and increased degeneration and fragmentation rates (**Figure** **2**A,B). In contrast, exposure to SMG from 8 to 14 h did not lead to significant effects, suggesting that the proper establishment of the MI stage is crucial for oocyte maturation under SMG conditions (Figure 2A,B).

The correct assembly of the spindle of the MI phase is the key to meiosis. To investigate this, MI‐stage oocytes were immunolabeled with α‐tubulin to assess spindle morphology and counterstained with Hoechst to examine chromosome arrangement (Figure 2E). In the NG group, MI‐stage oocytes displayed a typical barrel‐shaped spindle with chromosomes neatly aligned at the equator (Figure 2E, left). In contrast, SMG‐exposed oocytes showed a significantly higher incidence of abnormal spindle assembly (70.35±3.58%, Figure 2C, E). The abnormalities were primarily in the form of multipolar and wide‐polar spindles, with multipolar spindles increasing more than 26‐fold (from 0.83 ± 1.67 to 22.05 ± 2.74) and wide‐polar spindles increasing nearly fourfold (from 4.80 ± 1.25 to 18.51 ± 3.31) (mean ± SD) (Figure 2D, E). Additionally, the chromosome plate width was significantly greater in the SMG group (Figure 2F, G).

Under SMG conditions, most MI oocytes exhibited distorted spindle morphology characterized by multipolar or wide bipolar spindles, which are typically associated with defects in MTOC clustering. Under NG conditions, microtubules self‐organized into bipolar structures, with MTOC components clustering at the spindle poles (Figure 2H,I). Further quantitative analysis of the spatial distribution of MTOCs and chromatin revealed that, in NG oocytes, both signals were tightly clustered, with MTOCs symmetrically positioned on either side of the chromosomes (Figure 2J, top). However, SMG oocytes displayed various forms of aberrant MTOC distribution (Figure 2H), including unilateral loose clustering (Figure 2I, J, second row), dispersed localization (Figure 2I,J, third row), and spherical aggregation (Figure 2I,J, bottom). These aberrant MTOCs exhibited poor adhesion to the spindle ends and tended to detach from the spindle poles, suggesting that SMG impairs spindle assembly and delays MTOC aggregation, leading to disrupted oocyte maturation.

### 2.3 SMG Impairs KT‐MT Attachment, Aneuploidy, and Polarity in Oocytes

**Figure 3:**
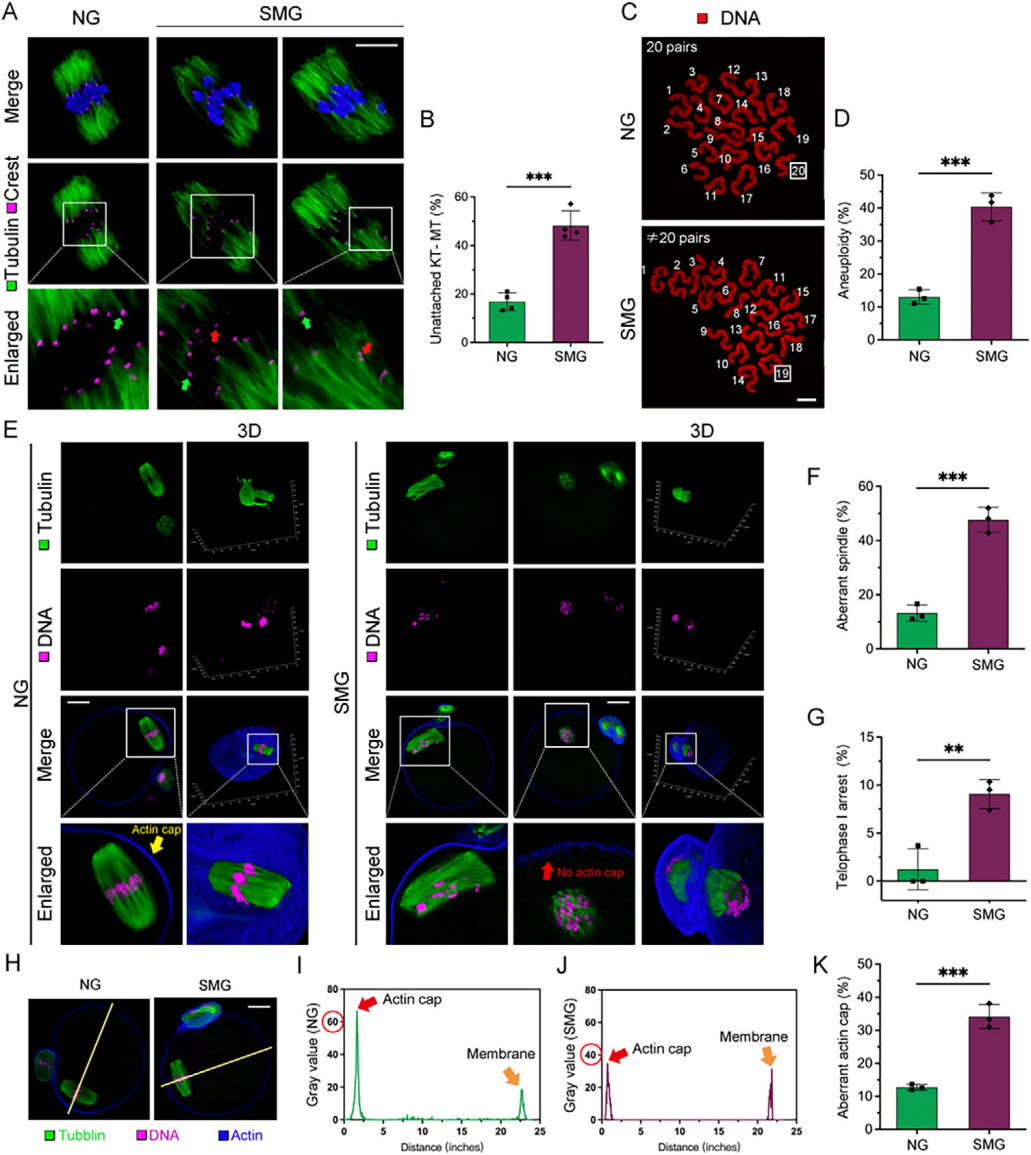
SMG on Kinetochore‐Microtubule (KT‐MT) Attachment, Aneuploidy, and Actin Polarity in Mouse Oocytes. A) Representative images of KT‐MT attachments in MI oocytes under NG and SMG conditions. Oocytes were incubated in M2 medium at 4°C for 10 min to induce depolymerization of unstable microtubules, followed by fixation and immunostaining for α‐tubulin (microtubules, green), CREST (kinetochores, carmine), and Hoechst (DNA, blue). Scale bar: 10 µm. B) Quantification of defective KT‐MT attachments in NG and SMG oocytes. C,D) Representative images of chromosome spreads from NG and SMG oocytes after 14 h of in vitro maturation and quantification of aneuploidy. Scale bar: 5 µm. E) Representative immunofluorescence images of α‐tubulin (microtubules, green) and phalloidin (actin, blue) in NG and SMG oocytes. Scale bar: 25 µm. F,G) Quantification of spindle abnormalities and telophase I arrest rates in MII‐stage oocytes under NG and SMG conditions. H–J) Profiles of phalloidin fluorescence intensity across the actin cap region in NG and SMG oocytes. The yellow line indicates the measurement axis perpendicular to the spindle length. Scale bar: 25 µm. K) Quantitative analysis of peak actin fluorescence intensity in NG and SMG oocytes with visible actin caps.

SMG‐exposed oocytes exhibited significantly more chromosome misalignments, indicating abnormalities in kinetochore‐microtubule (KT‐MT) attachment. To further investigate this, we used cold treatment to disaggregate unstable microtubules and labeled kinetochores with CREST and microtubules with anti‐α‐tubulin antibodies (**Figure** **3**A). In the NG group, most kinetochores were attached to microtubules, maintaining proper chromosome alignment. In contrast, the SMG group displayed a significantly higher proportion of unattached kinetochores (48.25 ± 6.05%; Figure 3A, B), indicating disrupted KT‐MT attachment and unstable chromosome bi‐orientation, which increases the risk of aneuploidy. To confirm this, we analyzed the karyotypes of MII oocytes via chromosome spreading (Figure 3C). Aneuploidy rates were significantly higher in SMG‐matured oocytes compared to the NG group (NG: 13.00%, n = 38; SMG: 40.38%, n = 42, *P <* 0.001; Figure 3D)

We observed entangled chromosome morphology in SMG‐exposed oocytes. To examine this further, MII‐stage oocytes were reconstructed in 3D using phalloidin to label actin and tubulin to label microtubules (Figure 3E). SMG‐exposed oocytes showed abnormal spindle assembly (Figure 3F) and a significantly increased rate of telophase I (TI) arrest (Figure 3G). The actin network, critical for spindle migration and anchoring,^[^ ^14^ ^]^ was severely affected under SMG conditions. ≈35% of SMG‐matured oocytes exhibited defective actin cap formation, a marked increase compared to the NG group (Figure 3H, K). Moreover, cortical actin signals were significantly reduced in the SMG group, actin was absent around the SCCs, indicating a loss of oocyte polarity (Figure 3I, J). In summary, SMG disrupts KT‐MT attachment, causes aneuploidy, and leads to polarity imbalance in MII oocytes, impairing their maturation and developmental potential.

### 2.4 SAC Activation is Intact Under SMG, and Prolonged Chromosome Division Stage Enhances PB1 Emission

**Figure 4:**
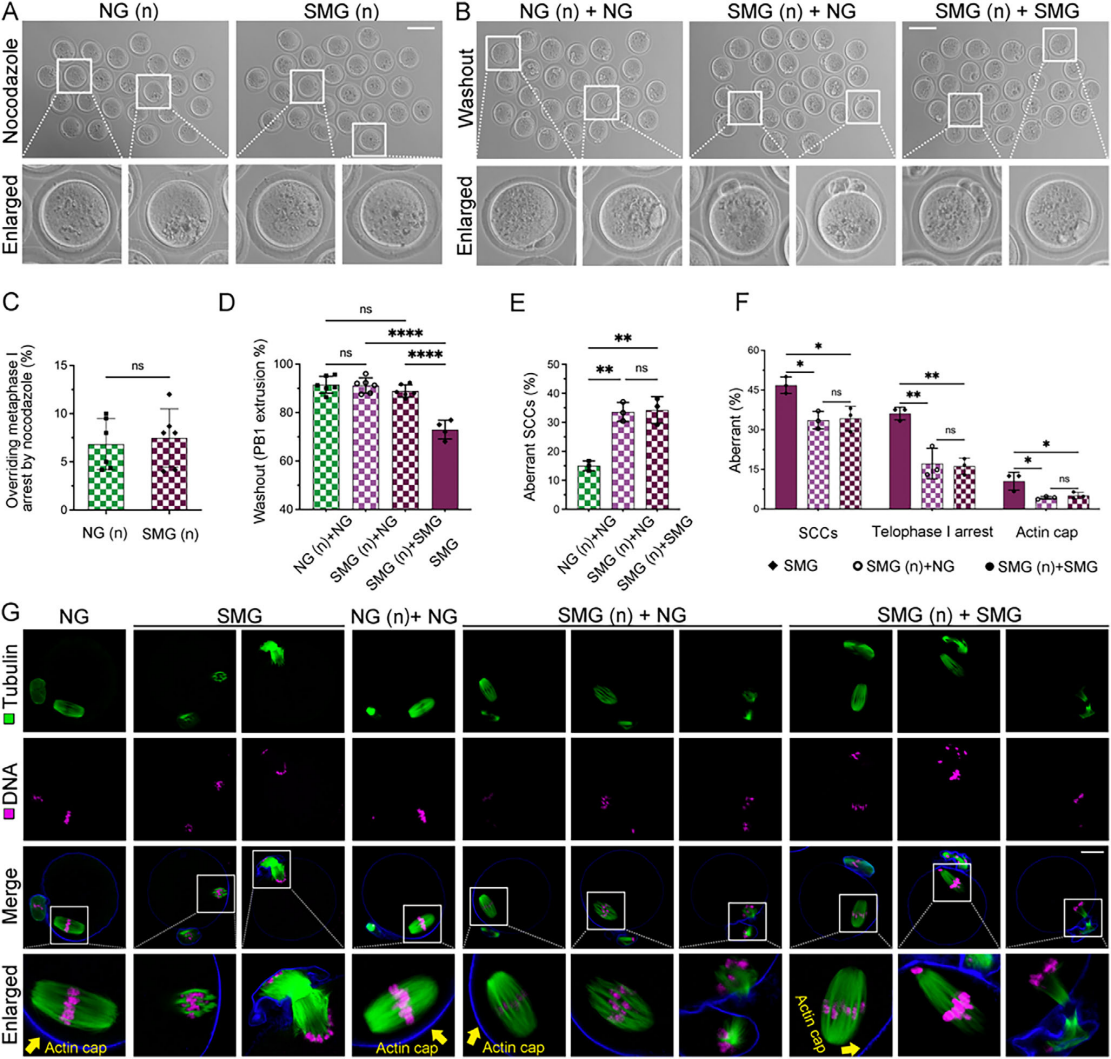
Effects of SMG on Oocytes Treated with Nocodazole. A) Representative images of MI oocytes matured for 12 h in the presence of 400 nM nocodazole. Scale bar: 100µm. B) Following nocodazole removal, oocytes were cultured for an additional 4 h, and PB1 extrusion was assessed under different culture conditions. n in parentheses indicates nocodazole treatment. C) Proportion of oocytes overriding MI arrest after nocodazole treatment in NG (n = 133) and SMG (n = 147) groups. D, E) Quantitative analysis of PB1 extrusion and spindle abnormalities following nocodazole removal in oocytes. F) Rates of spindle defects, telophase I arrest, and actin cap formation in SMG, SMG (n) + NG, and SMG (n) + SMG oocytes at the MII stage. n in parentheses indicates nocodazole treatment. G) Representative immunofluorescence images showing α‐tubulin (microtubules, green) and phalloidin (actin, blue) staining in oocytes under different culture conditions.

The accelerated meiotic progression under SMG raised the possibility that spindle assembly checkpoint (SAC) activity might be compromised. To evaluate SAC function, oocytes were matured and arrested at the MI stage in low concentration (400 nM/ml) of the spindle poison nocodazole. After 14 h of meiotic resumption, both NG and SMG groups remained arrested at MI, indicating that SAC activation was intact under SMG (**Figure** **4**A, C). Following nocodazole removal, oocytes were cultured for an additional 4 h under either NG or SMG conditions, and the PB1 emission rate was assessed (Figure 4B). In the NG group, PB1 emission exceeded 90%, unaffected by nocodazole treatment (Figure 4D). Notably, PB1 emission in the SMG (n) group, initially inhibited by nocodazole, returned to levels comparable to the NG group, significantly higher than in the SMG group without nocodazole treatment (Figure 4D). These findings suggest that extending the chromosome division stage by nocodazole treatment alleviates the negative effects of SMG on PB1 emission.

We further analyzed spindle morphology and actin distribution in mature oocytes following nocodazole treatment. Compared to the NG (n) group, the SMG (n) group exhibited a significantly higher incidence of abnormal meiotic spindle chromosome complex (SCC) (Figure 4E, G). However, in the nocodazole‐treated SMG group, the rate of SCC (n) abnormalities was significantly reduced, along with improved TI arrest and actin cap distribution, compared to the untreated SMG group (Figure 4F, G). Regardless of whether the oocytes were subsequently cultured under NG or SMG conditions, no significant differences in spindle morphology or polarity were observed in the nocodazole‐treated oocytes (Figure 4G). These results demonstrate that SAC activation remains functional under SMG, and prolonging the chromosome division stage by nocodazole treatment can partially alleviate the detrimental effects of SMG on oocyte maturation.

### 2.5 SMG Disrupts Mitochondrial Function, Leading to Increased Oxidative Phosphorylation and ATP Level in Oocytes

**Figure 5:**
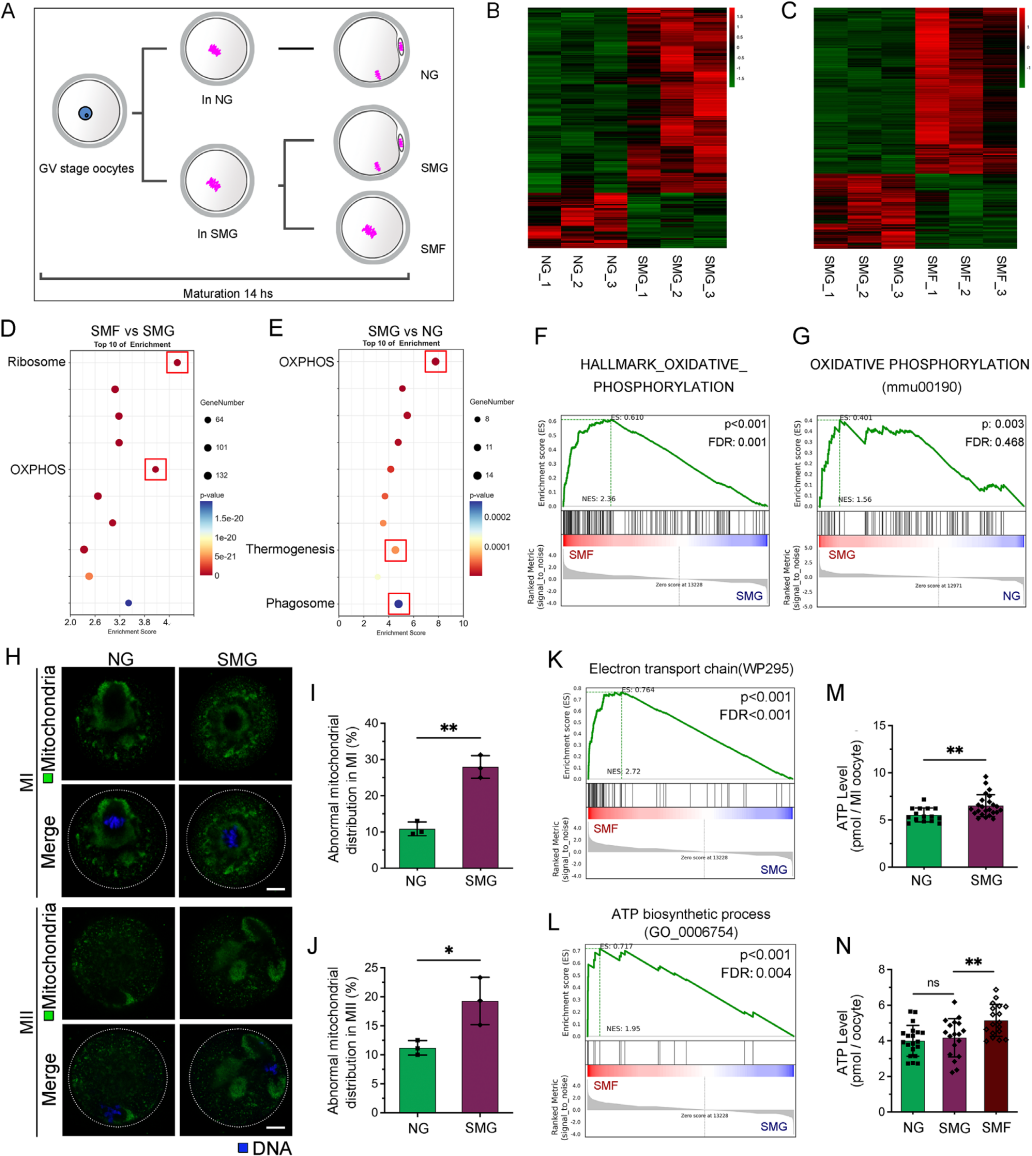
RNA Sequencing Indicates That SMG Leads to Increased Oxidative Phosphorylation and ATP Level in Oocytes. A) Schematic overview of oocyte collection for RNA‐seq from NG, SMG, and SMF groups. B, C) Heatmaps depicting differential gene expression in NG, SMG, and SMF oocytes. D, E) Kyoto Encyclopedia of Genes and Genomes (KEGG) enrichment analysis showing enriched pathways for differentially expressed genes in SMF vs. SMG and SMG vs. NG comparisons. F, G) Gene set enrichment analysis (GSEA) results for OXIDATIVE_PHOSPHORYLATION in SMF vs. SMG and SMG vs. NG groups. ES, Enrichment score; NES, normalized ES; FDR, false discovery rate. H) Representative images of mitochondrial distribution in NG and SMG oocytes at the MI (upper panels) and MII (lower panels) stages. Scale bar: 25 µm. I, J) Quantification of abnormal mitochondrial distribution in NG and SMG oocytes at the MI and MII stages, respectively. K) GSEA showing enrichment of the electron transport chain pathway (WP295) in SMF oocytes (NES = 2.72, FDR < 0.001). L) ATP biosynthetic process (GO:0006754) also enriched in SMF oocytes (NES = 1.95, FDR = 0.004). (M) ATP levels are significantly increased in MI‐stage oocytes under SMG (mean ± SEM; *p* < 0.01). (N) ATP content in MII oocytes shows a significant increase in SMF compared to SMG and NG (mean ± SEM; ns, not significant; *p* < 0.01).

To investigate the molecular mechanisms underlying oocyte maturation under simulated microgravity (SMG), we categorized oocytes into three groups: NG (normal gravity), SMG (matured under SMG), and SMF (failed to mature under SMG) (**Figure** **5**A). Smart‐seq2 analysis was performed to assess gene expression variations across these groups (Figure 5B, C). Compared to SMG‐matured oocytes, the SMF group exhibited extensive transcriptional changes, with 2098 genes upregulated and 947 downregulated (Figure S1B,C, Supporting Information). In SMG‐matured oocytes, 176 genes were upregulated, while 53 were downregulated relative to the NG group (Figure 1SB, D).

KEGG enrichment analysis revealed significant upregulation of genes associated with ribosomal function and oxidative phosphorylation (OXPHOS) in the SMF group (Figure 5D), suggesting a cellular stress response linked to mitochondrial dysfunction. In SMG‐matured oocytes, OXPHOS‐related genes also showed marked enrichment compared to NG oocytes, and this pathway exhibited the most prominent enrichment signal among the top ten terms (Figure 5E), highlighting the involvement of mitochondrial metabolism in the oocyte's response to microgravity. Gene set enrichment analysis (GSEA) further confirmed a marked disturbance in mitochondrial energy metabolism pathways in SMG‐exposed oocytes (Figure 5F, G). Specifically, HALLMARK_OXIDATIVE_PHOSPHORYLATION was significantly enriched in SMF compared to SMG (NES = 2.36, FDR = 0.001), supporting the notion that failed maturation is accompanied by enhanced mitochondrial activity. In the SMG versus NG comparison, the KEGG oxidative phosphorylation pathway (mmu00190) also showed enrichment (NES = 1.56, *p* = 0.003), though this did not reach statistical significance after FDR correction (FDR = 0.468), suggesting a possible trend toward increased oxidative metabolism. These results indicate that simulated microgravity perturbs mitochondrial energy metabolism, which may contribute to impaired oocyte maturation.

Since OXPHOS occurs within mitochondria, we further assessed mitochondrial distribution to explore potential alterations in mitochondrial function under SMG. Mitochondrial staining using MitoTracker revealed distinct distribution patterns at different meiotic stages (Figure 5H). In NG oocytes at the MI stage, mitochondria were concentrated around the spindle, providing energy for spindle assembly and chromosome segregation (Figure 5H, upper‐left panels). In contrast, SMG‐exposed oocytes showed abnormal mitochondrial clustering outside the spindle region (Figure 5H, upper‐right panels), with a significantly higher incidence of such misdistribution compared to NG oocytes (Figure 5H, I). At the MII stage, NG oocytes displayed a uniform mitochondrial distribution, indicative of energy balance and preparation for post‐fertilization development (Figure 5H, lower‐left panels). However, SMG‐matured oocytes exhibited abnormal mitochondrial clustering at the MII stage (Figure 5H, J).

To further validate the functional consequences of elevated OXPHOS activity, we examined ATP production, the key energetic output of mitochondrial metabolism.^[^ ^15^, ^16^ ^]^ GSEA indicated that the electron transport chain pathway (WikiPathways WP295) was significantly enriched in SMF oocytes (NES = 2.72, FDR < 0.001), confirming enhanced OXPHOS‐related transcriptional activity (Figure 5K). A moderate enrichment trend for the same pathway was also observed in SMG‐matured oocytes compared to NG (NES = 1.49, p = 0.022, FDR = 0.459; Figure S2A, Supporting Information). Additionally, the ATP biosynthetic process (GO:0006754) was significantly enriched in SMF oocytes (NES = 1.95, FDR = 0.004) but was not notably altered in the SMG group (Figure 5L).

Consistent with the transcriptomic evidence, direct measurement of ATP levels in MI‐stage oocytes revealed a significant increase under SMG conditions (Figure 5M). When ATP levels were assessed after 14 h of in vitro maturation across all groups, oocytes from the SMF group showed a marked increase compared to both NG and SMG groups (Figure 5N). Although SMG oocytes displayed a trend toward higher ATP content, the difference was not statistically significant relative to NG (Figure 5N). These results indicate that SMG induces a dysregulated elevation of ATP production, likely driven by mitochondrial overactivation, which may in turn impair oocyte maturation.

### 2.6 Overactivation of the Mitochondrial Unfolded Protein Response Impairs Oocyte Maturation and Disrupts Mitochondrial Genome–Encoded Gene Expression

**Figure 6:**
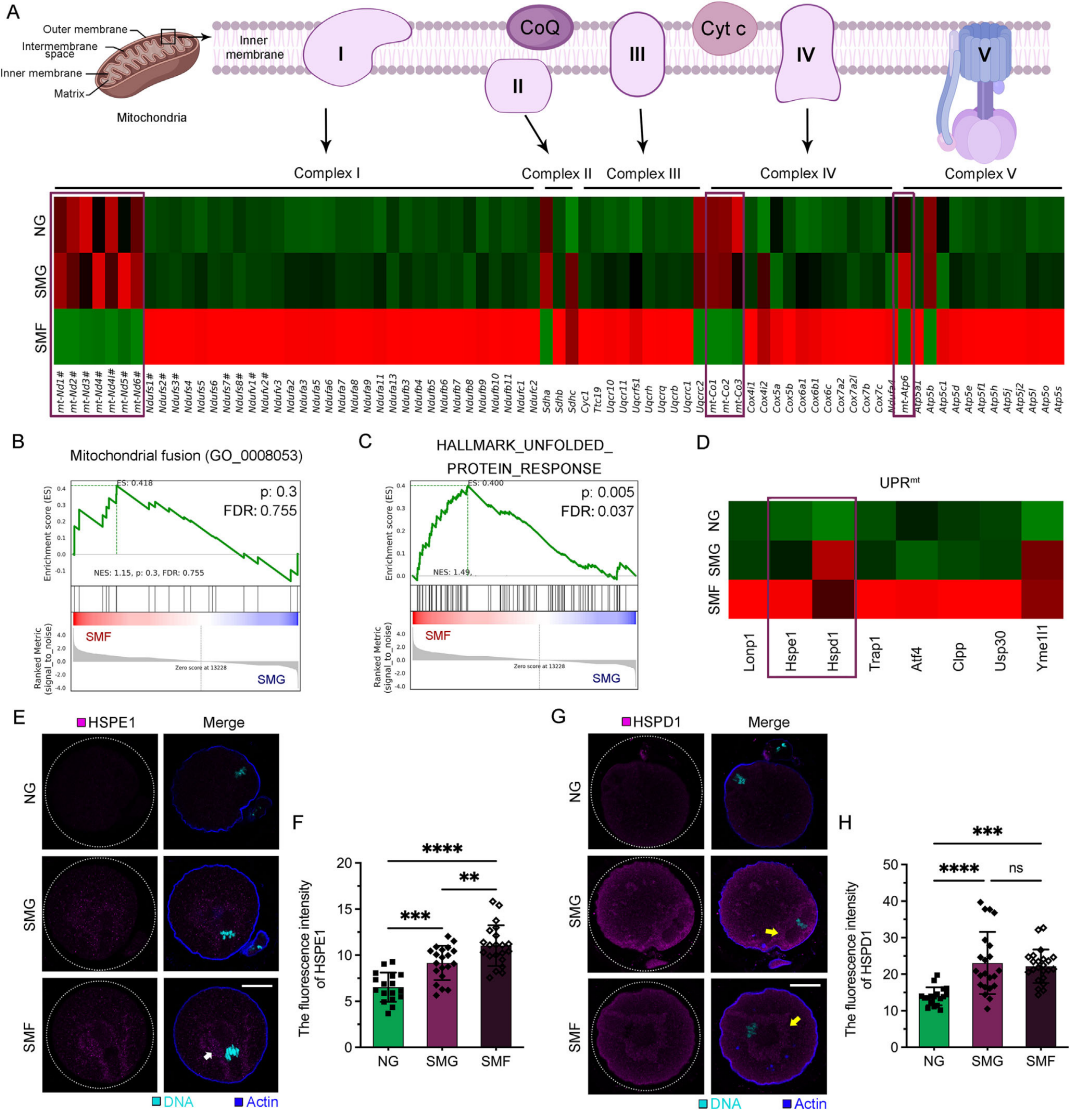
SMG disrupts mitochondrial gene expression and activates the UPR^mt^. A) Heatmap showing differential expression of oxidative phosphorylation (OXPHOS)‐related genes encoded by the mitochondrial (highlighted) and nuclear genomes across NG, SMG, and SMF oocytes. Core subunits of complex I are indicated with #. B) GSEA of the mitochondrial fusion pathway (GO:0008053) showing no significant enrichment in SMF versus SMG oocytes (NES = 1.15, FDR = 0.755). C) GSEA showing significant enrichment of the unfolded protein response (HALLMARK_UPR) in SMF oocytes (NES = 1.49, FDR = 0.037). D) Heatmap showing elevated expression of UPR^mt^ related genes in SMF oocytes. E, F) Immunofluorescence staining and quantification of HSPE1 in MII oocytes. (G, H) Immunofluorescence staining and quantification of HSPD1 in MII oocytes. Scale bar: 25 µm.

To further dissect the transcriptional changes underlying mitochondrial oxidative metabolism, we examined the expression of genes involved in OXPHOS, a process carried out by multiprotein complexes assembled from nuclear and mitochondrial DNA‐encoded subunits.^[^ ^17^, ^18^ ^]^ Heatmap analysis revealed that OXPHOS‐related genes were broadly upregulated in SMF oocytes, indicating elevated mitochondrial activity under failed maturation conditions (**Figure** **6**A). However, a notable exception was observed in mitochondrial genome–encoded OXPHOS subunits (e.g., *mt‐Nd4*, *mt‐Co2*, *mt‐Atp6*), which were consistently downregulated (Figure 6A, purple boxes), in contrast to the robust upregulation of their nuclear‐encoded counterparts.

To explore potential mechanisms underlying the suppression of mitochondrial genome–encoded OXPHOS genes, we first evaluated the transcription of genes associated with mitochondrial dynamics. Expression of *Drp1* and *Opa1*, which regulate mitochondrial fission and fusion, respectively, showed no significant difference between groups (data not shown). GSEA of the mitochondrial fusion pathway (GO:0008053) further confirmed the absence of enrichment in SMF versus SMG comparisons (NES = 1.15, FDR = 0.755, *p* = 0.3; Figure 6B). Given the lack of changes in mitochondrial dynamics, we next assessed whether mitochondrial proteostasis might be affected. GSEA revealed significant enrichment of the unfolded protein response (UPR) signature in SMF oocytes (NES = 1.49, FDR = 0.037; Figure 6C), implicating mitochondrial stress responses. Additionally, expression of key mitochondrial unfolded protein response (UPR^mt^) genes was elevated in SMF oocytes (Figure 6D).

To validate UPR^mt^ activation, we performed immunofluorescence staining and quantification of two key UPR^mt^ effectors: HSPE1 and HSPD1. HSPE1 expression was low and uniformly distributed in NG oocytes, moderately increased in SMG, and showed cortical aggregation with significantly higher intensity in SMF oocytes (Figure 6E,F). HSPD1 levels were elevated in both SMG and SMF groups compared to NG, and the protein localized around the meiotic spindle (Figure 6G,H). Notably, in SMF oocytes, HSPD1 is localized around abnormal, multipolar spindles, suggesting an association between mitochondrial stress and meiotic defects. In summary, SMG‐induced overactivation of OXPHOS in oocytes leads to maturation arrest, which is mediated by impaired mitochondrial gene expression through activation of the UPR^mt^.

The abnormalities in mitochondrial distribution, coupled with elevated OXPHOS activity and the overactivation of UPR^mt^, likely contribute to excessive reactive oxygen species (ROS) accumulation. ROS staining confirmed that SMG exposure induced greater oxidative stress in oocytes compared to NG controls (Figure S3A–C, Supporting Information), suggesting disrupted energy metabolism in oocytes matured under SMG. Furthermore, oocytes exposed to SMG exhibited reduced developmental potential (Figure S2C, Supporting Information).

### 2.7 SMG Disrupts Oocyte Maturation by Increasing Mitochondrial Membrane Potential

**Figure 7:**
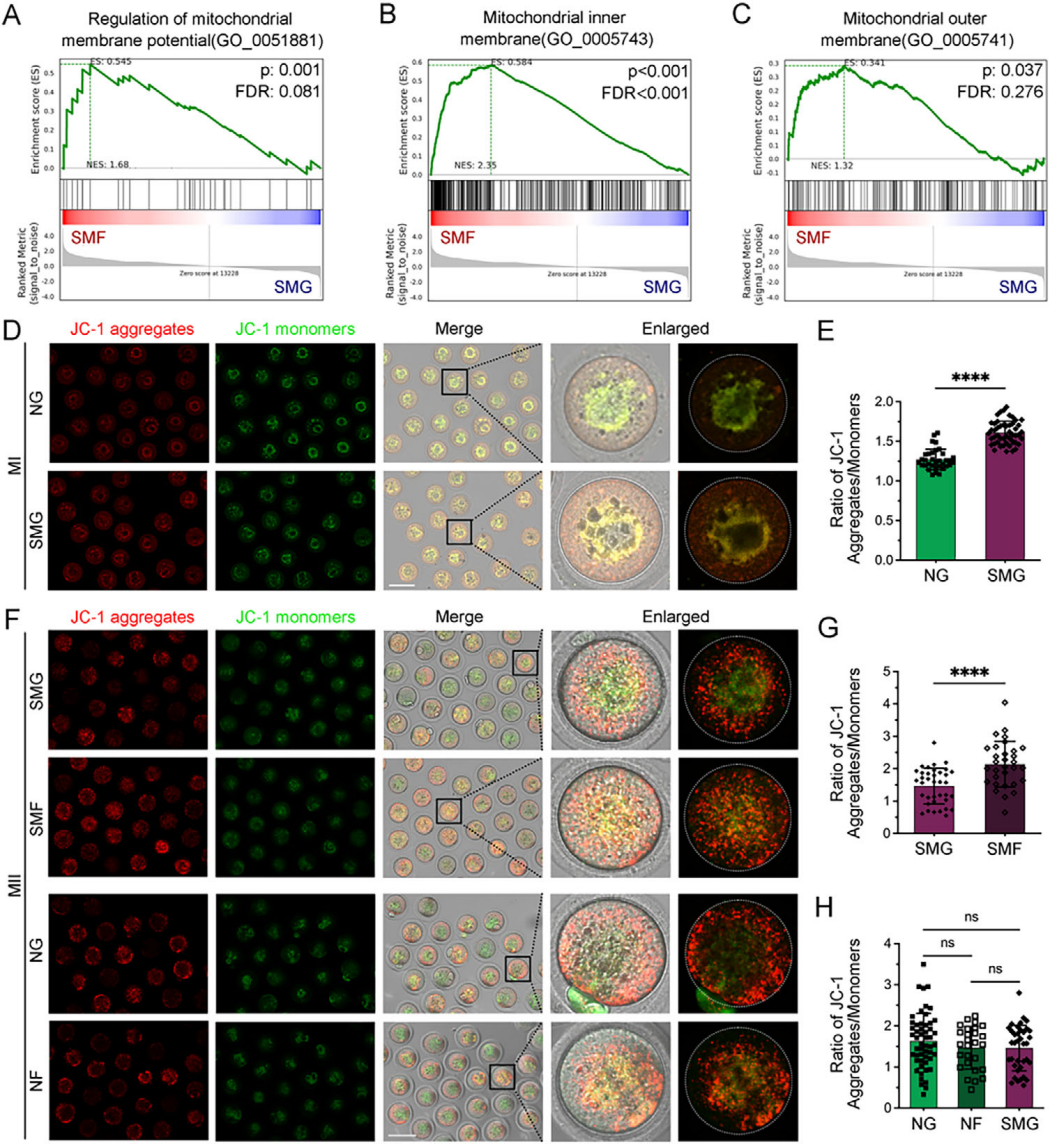
SMG Impairs Oocyte Maturation by Increasing Mitochondrial Membrane Potential (MMP). A–C) GSEA showing enrichment of gene sets related to mitochondrial membrane potential regulation (GO:0051881), mitochondrial inner membrane (GO:0005743), and outer membrane (GO:0005741) in SMF versus SMG oocytes. D) Representative images of JC‐1 staining in MI oocytes from NG and SMG groups, showing JC‐1 aggregates (red), monomers (green), and bright‐field images. Scale bar: 100 µm. E) Quantification of the JC‐1 aggregate‐to‐monomer intensity ratio in MI oocytes. F) Representative images of MMP in MII oocytes from SMG, SMF, NG, and NF groups. Scale bar: 100 µm. G) Quantification of MMP in MII oocytes comparing SMF vs. SMG. H) Quantification of MMP in MII oocytes among NG, NF, and SMG groups. NF: oocytes that failed to extrude PB1 under NG. SMF: oocytes that failed to extrude PB1 under SMG.

Mitochondrial membrane potential (MMP) is a critical driving force for proton‐driven ATP synthesis during OXPHOS. GSEA analysis revealed significant upregulation of MMP‐related genes in oocytes from the SMF group (NES = 1.68, *p* = 0.001, FDR = 0.081) (**Figure** **7**A). Additionally, genes associated with both the inner and outer mitochondrial membranes were upregulated (Figure 7B,C), suggesting a disruption in the energy metabolism network in oocytes that fail to mature.

To directly assess MMP in MI‐stage oocytes, we performed JC‐1 staining, which indicated a significant increase in MMP in SMG‐exposed oocytes (Figure 7D,E), likely contributing to enhanced ATP production. We also assessed MMP in MII‐stage oocytes, including a fourth group of NG oocytes that failed to extrude PB1 (NF) (Figure 7F). MMP was significantly higher in the SMF group compared to the SMG group (Figure 7G). In contrast, no significant difference was observed between the NG and NF groups (Figure 7H), while SMG oocytes exhibited a non‐significant downward trend relative to NG (Figure 7H). These results suggest that SMG leads to abnormal elevation of MMP, particularly in oocytes that fail to complete maturation. This alteration in MMP, together with increased ATP levels, contributing to disrupted meiotic progression and oocyte arrest under SMG conditions.

### 2.8 M‐Phase Prolongation Enhances Oocyte Maturation by Promoting MTOC Aggregation Under SMG

**Figure 8:**
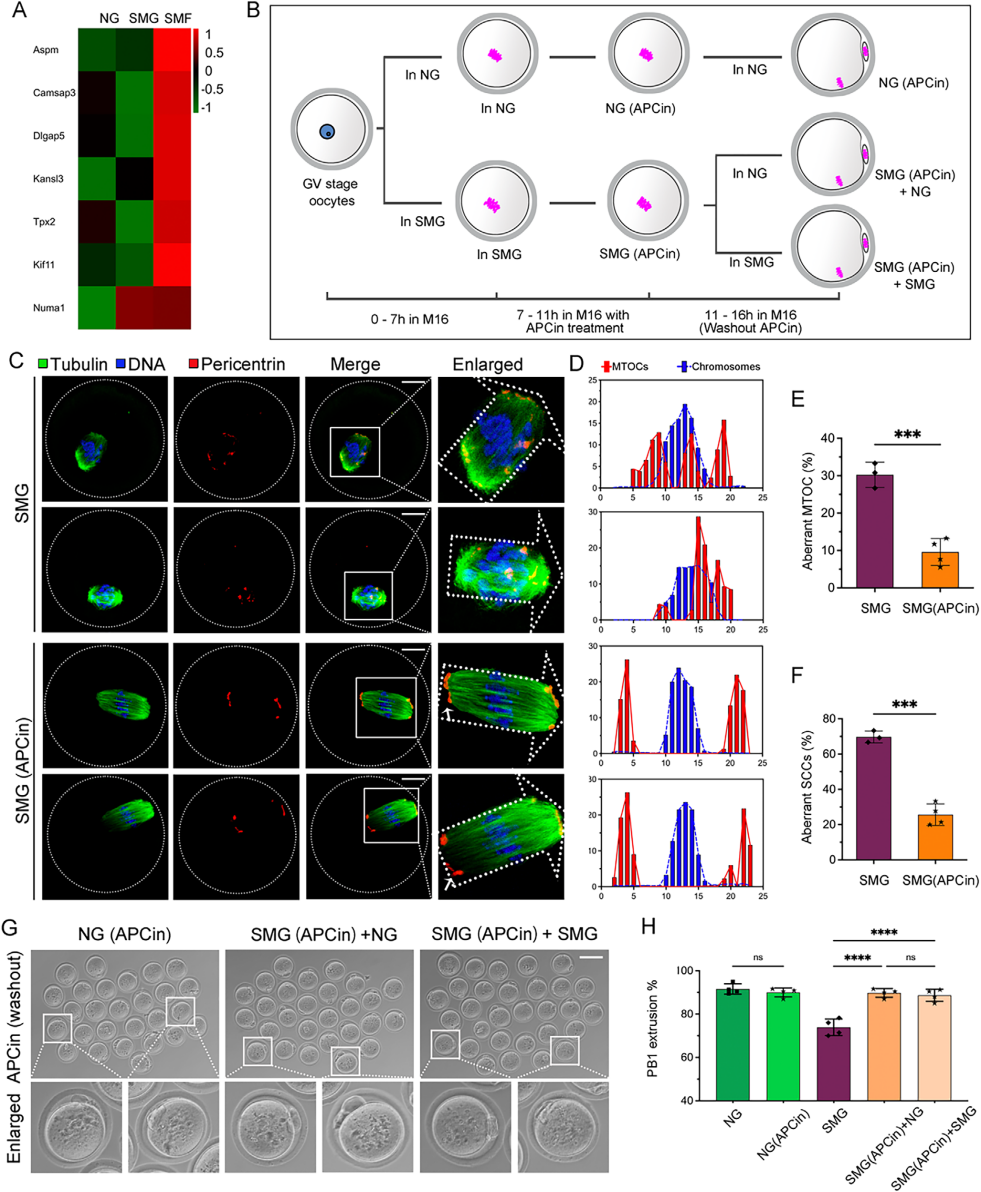
Prolonging M Phase Enhances Oocyte Maturation by Promoting MTOC Assembly Under SMG. A) Heatmap showing fold changes in the expression of MTOC regulation across NG, SMG, and SMF groups. B) Schematic of the experimental design. The MI phase was extended from 7 to 11 h using APCin, followed by APCin washout. Oocytes were then cultured under different gravity conditions for a total of 16 h. C) Immunofluorescence images of pericentrin (red) and microtubules (green) showing MTOC organization in MI‐stage oocytes from SMG and SMG (APCin) groups. Scale bar: 25 µm. D) Quantitative analysis of MTOC distribution along the spindle axis in MI oocytes. Pericentrin signals were binarized to calculate the individual MTOC area, and MTOC coordinates were plotted along the spindle axis (0‐25). MTOCs are shown in red; chromosomes in blue. E, F) Rates of MTOC abnormalities and abnormal meiotic spindle chromosome complex (SCC) rates recorded in MI‐stage oocytes from SMG and SMG (APCin) groups. G, H) Representative images and quantitative analysis of PB1 extrusion following APCin washout. Scale bar: 100 µm.

Our sequencing data revealed no significant downregulation of MTOC‐related genes under SMG conditions. Notably, we observed an upregulation of genes associated with MTOC fusion in oocytes that failed to mature under SMG (SMF), suggesting that the delayed MTOC fusion induced by SMG is not due to the downregulation of previously identified regulatory genes (**Figure** **8**A). GSEA further supported this finding, showing significant enrichment of MTOC‐related regulatory genes in SMF oocytes (Figure S2D, Supporting Information). These results indicate that supplementing functional proteins regulating MTOC through external interventions alone may not facilitate the faster fusion of MTOC under SMG conditions.

To identify strategies for improving oocyte maturation under SMG, we utilized APCin, a small molecule inhibitor of the anaphase‐promoting complex (APC), which reversibly extends the M phase during oocyte meiosis.^[^ ^19^, ^20^ ^]^ Since SMG accelerates meiotic progression, we prolonged the MI phase from 7 to 11 h using APCin (Figure 8B). We first examined MTOC distribution in MI‐stage oocytes (Figure 8C,D). Compared to SMG‐exposed oocytes cultured for 8 h, those treated with APCin (11‐h M phase) exhibited significantly improved MTOC aggregation, forming a more organized bipolar configuration (Figure 8C–E). Additionally, the rates of multipolar and wide polar spindles were significantly reduced (Figure 8F). These results indicate that extending the M phase allows sufficient time for MTOC aggregation, promoting proper bipolar spindle formation and improving meiotic SCC morphology.

Following APCin washout, oocytes were cultured for an additional 16 h under NG or SMG conditions to assess PB1 emission (Figure 8G). Statistical analysis showed that APCin treatment did not negatively affect PB1 emission rates. Notably, oocytes exposed to SMG with an extended M phase exhibited significantly improved PB1 emission rates, regardless of whether they were subsequently cultured under NG or SMG conditions, achieving results comparable to those observed in NG oocytes (Figure 8H). In summary, SMG accelerates meiotic progression, leading to abnormal spindle assembly. Prolonging the M phase provides sufficient time for MTOC aggregation and assembly, effectively mitigating oocyte maturation defects caused by SMG. These findings offer a potential strategy for improving oocyte quality in microgravity environments.

## 3 Discussion

Oocyte maturation is essential for female reproduction, as errors in meiotic progression can lead to aneuploidy and developmental failure. As space exploration advances, understanding how microgravity affects reproductive health has become increasingly important. In this study, we demonstrate that simulated microgravity (SMG) disrupts oocyte maturation by accelerating meiotic progression while delaying microtubule‐organizing center (MTOC) coalescence. This temporal mismatch compromises spindle assembly, leading to chromosome misalignment, increased aneuploidy, and reduced first polar body extrusion. Notably, the spindle assembly checkpoint (SAC) remained functional, suggesting that meiotic acceleration is driven not by SAC failure but by mitochondrial dysregulation.

We further show that SMG alters mitochondrial function, evidenced by increased oxidative phosphorylation (OXPHOS), hyperpolarized mitochondrial membrane potential (MMP), and elevated ATP levels. These mitochondrial responses are distinct from those triggered by other stressors and somatic cell types under SMG, underscoring the unique sensitivity of oocytes. Even oocytes that matured under SMG displayed loss of polarity and reduced developmental potential, consistent with defects in actin cytoskeleton organization and failure of actin cup formation. Importantly, prolonging metaphase I by inhibiting the anaphase‐promoting complex (APC) restored the timing of spindle assembly and MTOC aggregation, significantly improving maturation rates, suggesting a potential strategy to improve oocyte quality in microgravity environments.

These findings underscore the unique and complex effects of microgravity on oocytes, which are sensitive to alterations in cellular architecture and energy metabolism. While previous studies have reported that SMG impairs germinal vesicle breakdown (GVBD),^[^ ^21^ ^]^ we did not observe this reduction. This discrepancy may be due to differences in culture volume and equilibration efficiency, as our 10 mL system may have provided more stable gas exchange conditions. In our study, SMG‐exposed oocytes exhibited a significant increase in diameter and zona pellucida thickness. Additionally, parthenogenetic activation demonstrated reduced developmental potential in oocytes matured under SMG, reinforcing the notion that microgravity adversely affects oocyte quality. This result is consistent with the study by Miglietta et al. (2023) that SMG interferes with the morphological dynamics of human MII oocytes and may cause impaired fertilization ability in the later stage,^[^ ^22^ ^]^ which together confirms the unique sensitivity of oocytes to microgravity in space.

The SMG‐induced abnormalities in microtubule dynamics and chromosome segregation are consistent with previous findings, further validating that changes in gravity can affect cell cycle progression and cytoskeletal morphology.^[^ ^23^, ^24^, ^25^ ^]^ Under normal gravity, these processes are tightly regulated to ensure stable cell division. However, in SMG, the time constraints on microtubule assembly and MTOC coalescence led to wide poles, multipolar spindles, and defective chromosome segregation. These defects may be more pronounced in human oocytes, given their inherently unstable MTOC structures.^[^ ^26^, ^27^ ^]^ We also observed that the increased diameter of SMG‐exposed mouse oocytes correlated with abnormal actin distribution, and the failure of actin cup formation contributed to a loss of cell polarity. This aligns with previous research showing actin‐related gene downregulation in follicles exposed to SMG,^[^ ^12^ ^]^ suggesting that microgravity disrupts actin‐mediated chromosome migration.

Disruptions in meiotic progression likely destabilize KT‐MT attachments.^[^ ^28^, ^29^ ^]^ Our results confirmed that SMG increases the frequency of abnormal microtubule‐kinetochore attachment. Combined with abnormal spindle morphology and chromosome misalignment, it further leads to increased aneuploidy. SAC function is essential for ensuring proper KT‐MT attachment and accurate chromosome segregation.^[^ ^30^ ^]^ While accelerated meiosis is typically associated with SAC inactivation,^[^ ^31^ ^]^ our findings indicate that SAC remained active under SMG conditions, likely as a result of abnormal spindle morphology and disrupted KT‐MT attachments. Although first polar body (PB1) extrusion initiated earlier under SMG, the final extrusion rate was reduced, highlighting the incomplete maturation process.

Mitochondrial dysfunction plays a significant role in SMG‐induced meiotic defects. Elevated OXPHOS activity, associated with increased electron leakage during ATP transfer,^[^ ^32^, ^33^, ^34^ ^]^ and oxidative stress,^[^ ^35^ ^]^ is commonly observed in response to microgravity. Oxidative stress caused by microgravity has been reported in various cell types.^[^ ^36^, ^37^, ^38^, ^39^, ^40^ ^]^ Our results confirm that OXPHOS activity and ATP levels were significantly higher in MI oocytes under SMG, indicating altered energy metabolism. It also raises concerns about the broader implications of microgravity, such as its potential to accelerate the progression of OXPHOS‐sensitive cancers.^[^ ^41^, ^42^ ^]^ Our data suggest that SMG also activates the mitochondrial unfolded protein response (UPR^mt^), as evidenced by the significant upregulation of HSPE1 and HSPD1, which accumulated around the spindle region—potentially in response to mitochondrial mislocalization and protein folding stress. While OXPHOS activity increased, mitochondrial‐encoded OXPHOS genes were downregulated in SMF oocytes, likely due to UPR^mt^ overactivation in response to mitochondrial stress. Previous studies have shown that UPR^mt^ serves as a protective mechanism,^[^ ^43^, ^44^ ^]^ activated to restore protein homeostasis (proteostasis) following mitochondrial protein misfolding.^[^ ^44^ ^]^ However, prolonged UPR^mt^ activation has been associated with age‐related diseases, including cancer and neurodegeneration.^[^ ^45^, ^46^, ^47^ ^]^

Mitochondria are highly sensitive to environmental stressors, and as an environmental stressor, microgravity can activate mitochondrial stress responses (MSRs). When the stress from misfolded mitochondrial proteins is not alleviated, it can lead to cellular dysfunction and, ultimately, cell death, as observed in our study where oocyte maturation was arrested. In primordial follicles, high UPR^mt^ levels suppress OXPHOS activity to maintain a low‐energy state,^[^ ^48^ ^]^ but under SMG, elevated OXPHOS activity and the overactivation of UPR^mt^, which contributes to accelerate meiotic progression and impairs oocyte quality. We propose that the low OXPHOS state observed in primordial follicle oocytes represents a long‐term regulatory adaptation rather than a transient response. Furthermore, during primordial follicle activation and oocyte development, oocyte volume increases ≈300‐fold,^[^ ^49^ ^]^ and mitochondrial numbers expand to hundreds of thousands.^[^ ^50^ ^]^ Thus, we suggest that the transient reduction of mitochondrial‐encoded OXPHOS gene expression in the SMF group, coupled with the relatively small contribution of mitochondrial genome‐regulated genes to overall oxidative phosphorylation, prevents direct impairment of OXPHOS by UPR^mt^. Moreover, significant differences in mitochondrial structure and function occur during oocyte development,^[^ ^51^ ^]^ which may explain the potential for distinct OXPHOS states under high UPR^mt^ conditions.^[^ ^52^ ^]^

Given the maternal inheritance of mitochondria, SMG‐induced mitochondrial dysfunction may have transgenerational consequences.^[^ ^53^ ^]^ Our parthenogenetic activation experiments revealed compromised developmental potential in oocytes exposed to SMG, raising concerns about long‐term reproductive risks. Previous studies have demonstrated that elevated UPR^mt^ propagates or maintains harmful mitochondrial DNA (mtDNA),^[^ ^54^ ^]^ raising concerns about the potential transgenerational effects of mitochondrial defects in SMG‐exposed oocytes. Given that human oocyte maturation lasts much longer than in mice, persistent UPR^mt^ activation could further exacerbate OXPHOS dysfunction. Moreover, mitochondrial defects in oocytes have been linked to developmental diseases, such as cardiac abnormalities, diabetes mellitus, and deafness.^[^ ^55^, ^56^, ^57^ ^]^ Recent studies have shown that enhancing mitochondrial DNA expression in neurons can improve aging outcomes,^[^ ^58^ ^]^ raising the possibility that SMG‐induced downregulation of mitochondrial genes could accelerate aging. These findings highlight the need to investigate the impact of SMG on mitochondrial‐mediated transgenerational inheritance and long‐term reproductive health.

Interestingly, unlike most damage models that result in MMP reduction, SMG exposure led to MMP hyperpolarization in oocytes, highlighting the unique sensitivity of oocytes to microgravity.^[^ ^40^, ^59^, ^60^ ^]^ In our study, the elevated MMP was more pronounced in oocytes that failed to extrude PB1, but was absent under NG conditions, supporting a link between mitochondrial hyperpolarization and meiotic arrest. This aligns with previous findings showing that MFN2 overexpression enhances MMP around the spindle, contributing to meiotic arrest.^[^ ^61^ ^]^ Our Smart‐seq2 data showed elevated MFN2 expression in SMG‐exposed oocytes, consistent with this phenotype. Previous studies using oocyte‐specific deletion of Mfn2 (via ZP3‐Cre) reported no significant impact on ovulation or offspring numbers in mice,^[^ ^62^ ^]^ suggesting that elevated MFN2 expression may serve as a stress response marker rather than a functional driver. Notably, increased MFN2 mRNA stability in granulosa cells has been shown to delay cellular senescence,^[^ ^63^ ^]^ further highlighting cell‐type‐specific roles of MFN2 in the ovarian microenvironment. These findings provide mechanistic insight into how altered energy metabolism under SMG contributes to meiotic failure.

Previous studies have shown that prolonging M phase can improve the aneuploidy of senescent mouse oocytes due to the lag of chromosome separation.^[^ ^20^ ^]^ To mitigate the negative effects of SMG on oocytes, we extended M‐phase duration through APCin inhibition, providing additional time for MTOC coalescence and spindle assembly. This strategy reduced SMG‐induced defects, improved KT‐MT attachment, and enhanced PB1 extrusion. These findings suggest that extending M‐phase duration could be an effective approach to improving oocyte quality in microgravity environments. However, the specific protein or gene responsible for microgravity perception in oocytes remains unidentified, which presents an important area for future research. Additionally, interspecies differences in oocyte maturation processes must be considered. For instance, KIFC1 contributes to greater spindle stability in mouse oocytes compared to human oocytes, which may influence the severity of microgravity‐induced defects.^[^ ^27^ ^]^ At the same time, it further indicates that the elevated energy metabolism state induced by SMG accelerates meiotic progression, which is the core of oocyte arrest, and that the damage caused by SMG can be rescued when sufficient time is given.

In conclusion, this study provides novel insights into the mechanisms underlying SMG‐induced oocyte maturation arrest. Specifically, SMG accelerates meiotic progression, increases OXPHOS and MMP, and compresses the time available for spindle assembly and MTOC coalescence, leading to abnormal spindle formation and meiotic arrest, ultimately reducing developmental potential. Extending M‐phase mitigated these defects, suggesting that modulating meiotic progression may be a viable strategy to improve oocyte quality in space environments. Future studies should focus on validating these findings in human oocytes, exploring pharmacological interventions targeting M‐phase regulation, and investigating the transgenerational consequences of SMG‐induced mitochondrial dysfunction in spaceflight models. These efforts will be critical for ensuring reproductive health in space exploration and beyond.

## 4 Experimental Section

### Animals

Female C57BL/6J mice (21 days old) were obtained from the Guangdong Medical Laboratory Animal Center (Guangdong, China). Animal care followed the Guide for the Care and Use of Laboratory Animals in Guangdong Province and was approved by the Ethics Committee at the Shenzhen Institutes of Advanced Technology (Approval Number: SIAT‐IACUC‐210427‐YYS‐LXH‐A1514‐01).

### Oocyte Collection, Culture, and Parthenogenetic Activation

Female mice were injected with 5 IU of pregnant mare serum gonadotropin (PMSG, Ningbo San Sheng Biotech, China) and sacrificed by cervical dislocation 44–48 h post‐injection. Fully grown oocytes arrested at the prophase of meiosis I were isolated from ovaries in M2 medium (Sigma‐Aldrich, USA) containing 2.5 µm milrinone (MCE, USA) to inhibit germinal vesicle breakdown (GVBD). Only oocytes displaying intact germinal vesicles were selected for further culture in M16 medium (Sigma‐Aldrich) under mineral oil at 37°C in a 5% CO_2_ atmosphere.

For simulated microgravity (SMG) conditions, oocytes were cultured using a rotating cell culture system (RCCS‐4D, SYNTHECON, USA), which simulates microgravity by reducing shear forces and creating a dynamic suspension environment.^[^ ^64^, ^65^ ^]^ The optimal rotation rate for inducing SMG was set at 15 rotations per min, based on previous studies.^[^ ^6^, ^66^, ^67^ ^]^ The oocytes were inoculated into 10 mL M16 of the culture vessel using the RCCS (SMG), cultured statically (NG‐RCV), and in medium covered with paraffin oil in normal gravity (NG).

Parthenogenetic activation was induced by placing in vitro matured oocytes in Ca^2^⁺‐free CZB medium (modified BMOC 2 medium) supplemented with 5 mM SrCl_2_ (10025 70–4; Sangon Biotech, China) 5 mg mL^−1^ cytochalasin B (CB) for 6 h. Activated oocytes were then cultured in KSOM medium, and the development of two‐cell embryos and blastocysts was assessed at 24 and 96 h, respectively.

### Immunofluorescence and Confocal Microscopy

Oocytes were fixed in 4% paraformaldehyde for 30 min, followed by permeabilization in 0.5% Triton X‐100 for 2 h. Samples were blocked in PBS containing 1% BSA, 0.1% Tween‐20, and 0.01% Triton X‐100 for 1 h and then incubated overnight at 4°C with primary antibodies: human anti‐centromere (1:500, Antibodies Incorporated 15‐234‐0001), mouse anti‐tubulin (1:300, Sigma, F2168), rabbit anti‐tubulin (1:500, Abcam ab6046), mouse anti‐pericentrin (1:400, BD Biosciences 611814), rabbit anti‐HSPE1 (1:300, Sigma–Aldrich ZRB1145), rabbit anti‐HSPD1 (1:300, Sigma‐Aldrich SAB4501465) and mouse anti‐HSPD1 (1:300, Absea KC‐6176). Secondary antibodies included Alexa Fluor 488 donkey anti‐mouse (1:500, Jackson 715‐545‐151), Alexa Fluor 568 donkey anti‐rabbit (1:500, Jackson 711‐025‐152), and Alexa Fluor 647 donkey anti‐human (1:500, Jackson 709‐605‐149). DNA was stained with Hoechst 33342 (10 µg mL^−1^, Sigma–Aldrich). F‐actin was visualized using Actin‐Tracker Red‐555 (1:200, Beyotime C2203S) and Actin‐Tracker Red‐488 (1:200, Beyotime C2201S). Fluorescence was analyzed under a confocal microscope (Leica sp8).

Z‐projections of pericentrin‐stained oocytes were used to quantify microtubule organizing centers (MTOCs) signals along the spindle axis. The distribution of MTOC and chromosomes was analyzed using ImageJ software (MacBiophotonics).

### Chromosome Spreading and Immunofluorescence

Metaphase II (MII) oocytes were collected after treatment with or without SMG and subjected to zona pellucida removal using acidic Tyrode's solution (Sigma–Aldrich). Zona‐free oocytes were fixed on glass slides in 1% paraformaldehyde containing 0.15% Triton X‐100 and 3 mM dithiothreitol (Sigma‐Aldrich) for air drying. DNA was stained with Hoechst 33342 and examined with a confocal microscope (Leica sp8).

### Nocodazole Treatment

To induce metaphase I (MI) arrest, oocytes from both NG and SMG groups were cultured in M16 medium containing 400 nM nocodazole (Sigma) dissolved in dimethyl sulfoxide (DMSO, Sigma) as previously described.^[^ ^68^ ^]^ Oocytes were imaged using bright‐field microscopy at 14 h post‐resumption of meiosis to assess PB1 extrusion. Following nocodazole removal, oocytes were cultured for an additional 4 h under either NG or SMG conditions, and the ability of the cells to progress to MII was evaluated.

### Library Preparation, Construction, and Sequencing

Total RNA from oocytes (30 oocytes per tube) was extracted using RNA lysis reagents and reverse‐transcribed into first‐strand cDNA using the Smart‐Seq2 protocol.^[^ ^69^ ^]^ The second‐strand cDNA was synthesized by PCR amplification. The cDNA library was constructed through fragmentation, end‐repair, and addition of a single adenine base at the 3ʹ end. Library quality was assessed using the Agilent Bioanalyzer 2100 system (Agilent Technologies, USA), and concentration was quantified using the StepOnePlus Real‐Time PCR System (Library valid concentration > 2 nM). Sequencing was performed on the Illumina HiSeq 2500 platform, generating 125 bp paired‐end reads.

### Filtering and Alignment of Sequencing Reads

Raw sequencing reads were filtered using a Perl‐based quality control pipeline. Trimmed reads shorter than 30 bp, reads containing adapter contamination, or those with a Phred quality score ≤19 in more than 15% of bases were removed. Reads with more than 5% N bases were also discarded. Filtered reads were mapped to the mouse reference genome using HISAT2 (v2.1.0).^[^ ^70^ ^]^ Mapped reads were visualized using the Integrative Genomics Viewer (IGV). Gene expression levels were calculated using HTSeq (v0.6.0), and fragments per kilobase million (FPKM) values were used to estimate gene expression.

### Differential Gene Expression and Functional Enrichment Analysis

Differentially expressed genes (DEGs) were identified using DESeq2 (v1.6.3), with statistical significance determined by the Wald test and adjusted using the Benjamini‐Hochberg method. Genes with a |log_2_(fold change)| ≥1.5 and an adjusted p‐value ≤0.05 were considered significant. Functional annotation was conducted using the DAVID tool (v6.7) to perform Gene Ontology (GO) and Kyoto Encyclopedia of Genes and Genomes (KEGG) pathway analyses.

### Mitochondrial Membrane Potential and Distribution

Mitochondrial membrane potential (ΔΨm) was assessed using the JC‐1 dye (1:200, Beyotime Biotech). Oocytes were incubated in M16 medium containing JC‐1 for 20 min, and fluorescence intensities were measured by confocal microscopy as previously described.^[^ ^71^ ^]^ The ΔΨm was calculated as the ratio of red to green fluorescence intensities.

Mitochondrial distribution was analyzed by incubating MI or MII oocytes in M16 medium containing 500 nM MitoTracker Green (Thermo Fisher) for 30 min. Labeled oocytes were mounted on glass slides and observed using a laser scanning confocal microscope (Leica sp8).

### ROS Assay

Reactive oxygen species (ROS) levels in MII oocytes were measured using 10 mM DCFH‐DA. Oocytes were incubated in M16 medium containing DCFH‐DA for 30 min, and fluorescence intensities were measured using confocal microscopy.

### Statistical Analysis

Data are presented as mean ± standard deviation (SD). Statistical analyses were performed using data from at least three independent biological replicates. Nonparametric Kruskal–Wallis tests were used to compare chromosome and kinetochore morphologies, PB1 extrusion, spindle defects, mitochondrial distribution, ΔΨm, ROS levels, and mitochondria‐related gene expression with Prism 9 software (GraphPad, USA). P values were designated as *P < 0.05, **P < 0.01, and ***P < 0.001.

## Conflict of Interest

The authors declare no conflict of interest.

## Author Contributions

Conceptualization: L.G., J.V.Z., and X.H.L. Methodology: L.G., Y.Q.G. Investigation: L.G., Y.Q.G., Y.L.Y., D.F.F., and C.Y.M. Visualization: L.G., and Y.Q.G. Supervision: J.V.Z., and X.H.L. Writing—original draft: L.G., and Y.Q.G. Writing—review & editing: J.V.Z., X.H.L., Y.Q.G., and L.G. Funding: J.V.Z., and X.H.L.

## Supporting information



Supporting Information

## Data Availability

The data that support the findings of this study are available from the corresponding author upon reasonable request.
