# Supplementary material for: The Critical Role of Enhanced OXPHOS and Mitochondrial Hyperpolarization in Simulated Microgravity‐Induced Oocyte Maturation Arrest
Source: Adv Sci (Weinh). 2025 Jul 18;12(38):e05570. doi: 10.1002/advs.202505570 (PMC12520490; doi:10.1002/advs.202505570)
Supplement: Supplementary file 1 — Supporting Information [file ADVS-12-e05570-s001.docx]

The Critical Role of Enhanced OXPHOS and Mitochondrial Hyperpolarization in Simulated Microgravity-Induced Oocyte Maturation Arrest

Lei Ge, Yuqing Gao, Feifei Du, Chiyuan Ma, Tianxia Xiao, Yali Yang^*^, Xiaohua Lei^*^, Jian V. Zhang^*^

**Supplementary Materials:**


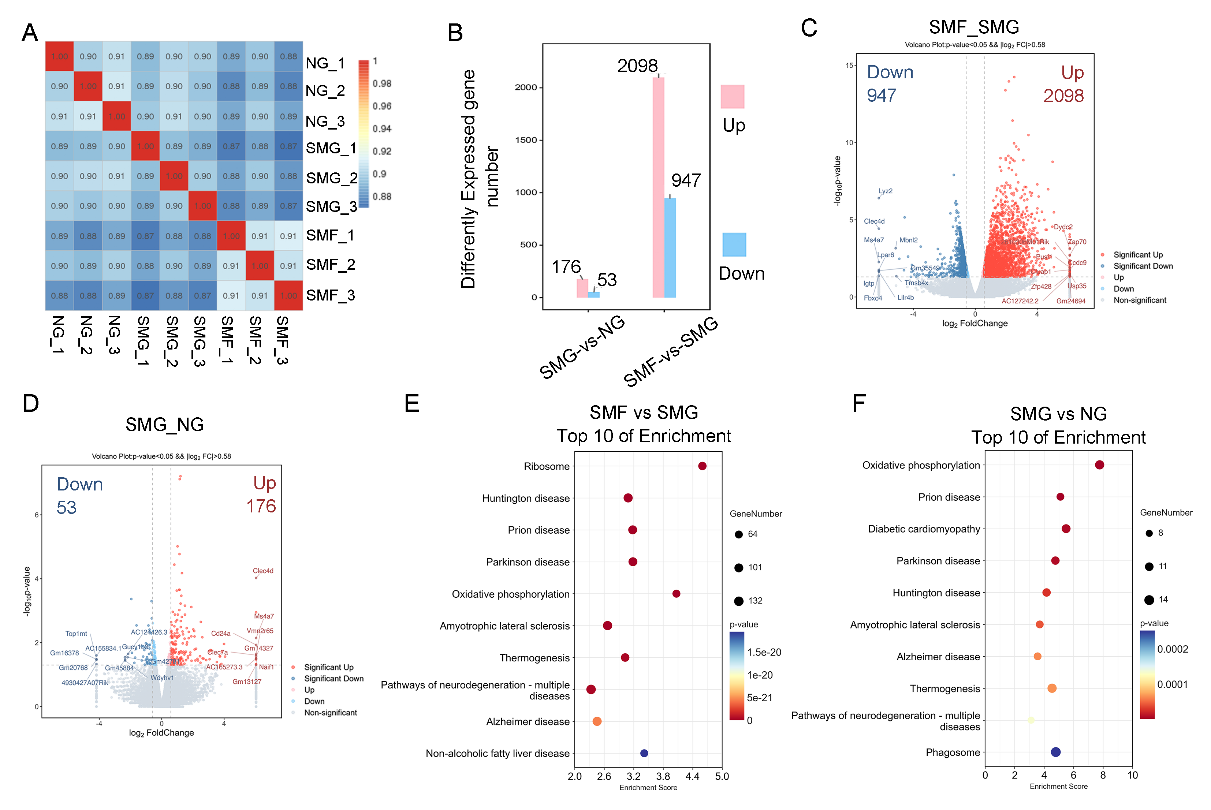


**Figure S1. RNA Sequencing Analyses of Oocytes from NG, SMG, and SMF Groups.**

(A) Heatmap of Spearman correlation coefficients showing transcriptomic similarities among oocytes from NG, SMG and SMF oocytes. (B) Total number of upregulated and downregulated genes in SMG vs. NG and SMF vs. SMG comparisons. (C, D) Volcano plots depicting transcriptional changes in SMF vs. SMG and SMG vs. NG groups. Genes with more than 1.5-fold changes in expression are highlighted in red (upregulated) and blue (downregulated). (E, F) KEGG enrichment analysis showing rich factors for differentially expressed genes in SMF vs. SMG and SMG vs. NG comparisons.

**
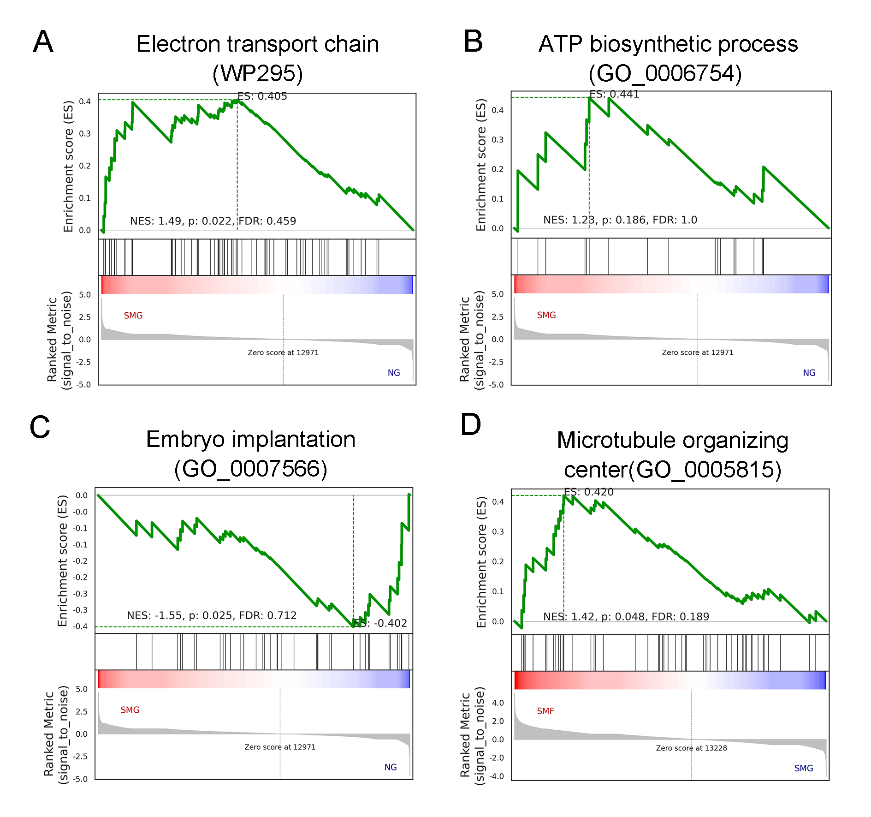
**

**Figure S2. GSEA Analyses of Oocytes from NG, SMG, and SMF Groups**

(A) The electron transport chain pathway (WP295) showed moderate enrichment in SMG oocytes compared to NG (NES = 1.49, *p* = 0.022, FDR = 0.459). (B) No significant enrichment was observed in the ATP biosynthetic process pathway (GO:0006754) in SMG oocytes (NES = 1.23, FDR = 1.0). (C) The embryo implantation pathway (GO:0007566) was negatively enriched in SMG oocytes (NES = –1.55, FDR = 0.712). (D) The microtubule organizing center pathway (GO:0005815) showed enrichment in SMF oocytes (NES = 1.42, *p* = 0.048, FDR = 0.189).

**
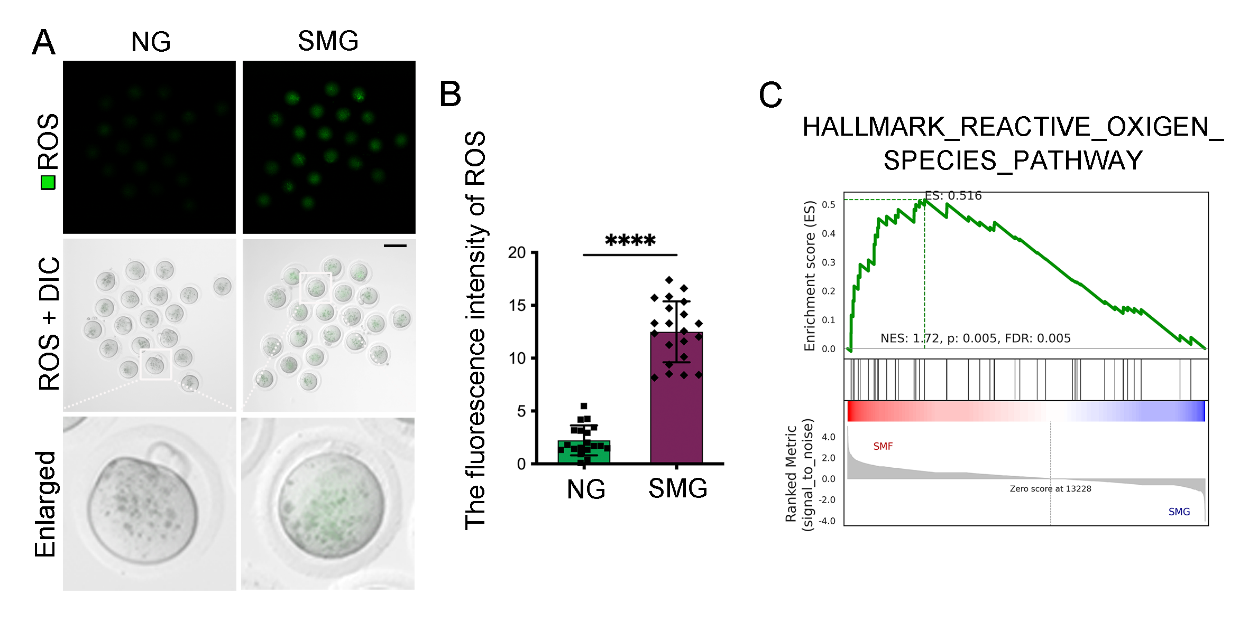
**

**Figure S3. SMG Exposure Increases Oxidative Stress in Oocytes**

(A) Representative images of DCFH-DA fluorescence showing reactive oxygen species (ROS) levels in NG and SMG oocytes. Scale bar, 100 μm. (B) Quantification of ROS fluorescence intensity (*p* < 0.0001). (C) GSEA showing significant enrichment of the HALLMARK_REACTIVE_OXYGEN_SPECIES_PATHWAY in SMF oocytes (NES = 1.72, *p* = 0.005, FDR = 0.005).
